# ABA-induced alternative splicing drives transcriptomic reprogramming for drought tolerance in barley

**DOI:** 10.1186/s12870-025-06485-y

**Published:** 2025-04-08

**Authors:** Anna Collin, Hubert Matkowski, Ewa Sybilska, Asmarany Biantari, Oliwia Król, Agata Daszkowska-Golec

**Affiliations:** Present Address: Institute of Biology, Biotechnology and Environmental Protection, Faculty of Natural Sciences, University of Silesia in Katowice, Jagiellońska 28, 40 - 032 Katowice, Poland

**Keywords:** Abscisic acid, Barley, Photosynthesis, Priming, Splicing

## Abstract

**Background:**

Abscisic acid (ABA) is a phytohormone that mediates plant responses to drought stress by regulating stomatal conductance, gene expression, and photosynthetic efficiency. Although ABA-induced stress priming has shown the potential to improve drought tolerance, the molecular mechanisms underlying ABA pretreatment effects remain poorly understood. This study aimed to determine how ABA pre-treatment at the booting stage influences physiological and molecular responses to drought at the heading stage in barley.

**Results:**

The ABA-treated plants exhibited earlier stomatal closure, increased expression of ABA-responsive genes (*HvNCED1*, *HvBG8*, and *HvA22*), and maintained higher chlorophyll levels under drought conditions. Photosynthetic parameters, including photosystem II activity, electron transport rate, and the number of active reaction centers, were preserved in ABA-pretreated plants compared with drought-only plants. Transcriptomic analysis revealed that ABA pre-treatment primed plants for faster activation of stress-responsive pathways, with enhanced expression of genes related to chromatin modifications, RNA metabolism, and ABA signaling during drought. Importantly, Alternative splicing (AS) and isoform switching were significantly amplified in ABA-pretreated plants, underscoring a unique molecular mechanism of ABA priming that enhances drought resilience. Post-stress recovery analysis revealed a greater number of differentially expressed genes (DEGs) and alternatively spliced transcripts (DAS) in ABA-pretreated plants, particularly those involved in chromatin organization and photosynthesis. Physiological analyses demonstrated that time- and dose-optimized ABA applications improved yield parameters, including grain weight and seed area, while mitigating spike sterility under drought conditions.

**Conclusions:**

This study demonstrates that ABA pretreatment enhances drought resilience in barley by triggering early stomatal closure, preserving chlorophyll content, and maintaining photosynthetic performance under water stress. At the molecular level, ABA priming accelerates stress-response pathways, promoting alternative splicing, isoform switching, and chromatin modifications that enable transcriptome plasticity. These processes facilitate faster recovery and sustain critical yield components, such as spike number and grain weight, when ABA is applied at optimized timing and concentrations. While large-scale ABA application poses challenges, this study provides a framework for breeding and agronomic strategies to mimic ABA effects, offering a practical path to enhance drought tolerance and yield stability in barley.

**Supplementary Information:**

The online version contains supplementary material available at 10.1186/s12870-025-06485-y.

## Introduction

Abscisic acid (ABA) is a key phytohormone that regulates plant responses to abiotic stresses, such as drought, by coordinating stomatal closure, gene expression, and physiological adaptations. ABA signaling is mediated through core components, including PYRABACTIN RESISTANCE PROTEINS/PYR-LIKE PROTEINS/REGULATORY COMPONENTS OF ABA RECEPTOR (PYR/PYL/RCAR) receptors, PHOSPHATASE 2 C (PP2 C) phosphatases, and SNF1-RELATED PROTEIN KINASE 2 (SnRK2) kinases, which activate ABA-dependent transcription factors, leading to the expression of stress-responsive genes [10, 38, 48, 54]. While ABA signaling plays a pivotal role in adaptation to unfavorable environments, its prolonged activation can impair photosynthesis, inhibit growth, and negatively impact seed development, leading to significant yield losses [73],Kavi [28]. ABA levels must therefore be carefully balanced to achieve drought tolerance without compromising growth. Low basal ABA levels support processes critical for growth and development, such as chloroplast biogenesis, cuticle formation, and xylem differentiation [7, 28]. In contrast, transient increases in ABA promote drought tolerance by inducing stomatal closure and stress-adaptation mechanisms [12, 50]. However, excessively high ABA levels can inhibit growth and long-term stress adaptation [56]. These findings highlight the potential of manipulating ABA levels as a strategy to achieve drought resilience in crops. Drought stress remains a major threat to global crop production, driven by increasing temperatures and reduced rainfall. Research suggests that pre-applying abiotic stress mitigators, such as ABA, polyamines, and nutrients, can prime plants for improved stress tolerance [3, 44],H. [75],[69]. For instance, ABA priming has been shown to enhance drought resilience in wheat, maize, and barley by improving water status, photosynthetic efficiency, and antioxidant defense system [60, 61, 67],[70]. In field-grown wheat, higher water content, improved photosynthesis, increased chlorophyll content, and a more efficient antioxidant system under drought conditions resulted in improved yield. ABA-treated maize (*Zea mays*) showed a better water status and increased catalase activity in response to drought at the pre-flowering stage, which ensured better grain yield [61]. Moreover, application of ABA to 30-d-old barley (*Hordeum vulgare*) resulted in higher chlorophyll content, better photosynthesis parameters, and higher activity of antioxidant enzymes under drought [60]. ABA pretreatment increases endogenous ABA content, which activates ABA-dependent transcription factors and stress-responsive genes and maintains their content/expression at a higher level than usually observed. These studies indicate that ABA priming can serve as a cost-effective strategy to improve cereal tolerance to drought. Generally, stress priming evokes epigenetic changes such as methylation and acetylation, especially in histones. Histone modification also contributes to higher activation of stress-responsive genes [11, 35, 69]. Additionally, priming can affect the expression of genes regulating plant development; for example, mild drought applied before flowering changes the expression of genes encoding transcription factors involved in flowering transition and inflorescence patterning in maize [17].

Given the critical role of ABA in stress responses, we hypothesized that ABA pretreatment at the booting stage would prime barley plants for enhanced drought resilience at the heading stage. Barley (*Hordeum vulgare*), a globally cultivated cereal with high abiotic stress tolerance, serves as an excellent model for investigating drought adaptation mechanisms at the molecular level [13, 26, 40, 43]. To test our hypothesis, we applied ABA and drought treatments in a controlled experimental design and investigated physiological and transcriptomic responses. Our findings provide novel insights into ABA-induced priming mechanisms and highlight its potential for improving crop drought resilience.

Moreover, ABA pretreatment may provide a'head start'in the recovery process, allowing the plant to rebuild and return to regular growth sooner after the drought stress is alleviated. Overall, changes in gene expression could help ABA-pretreated plants to manage drought stress more effectively, potentially leading to better survival or recovery than non-pretreated plants. These findings advocate for the exploration of genotypes with moderately elevated ABA levels for improved drought resilience.

## Material and Methods

### Experimental setup

**Fig. 1:**
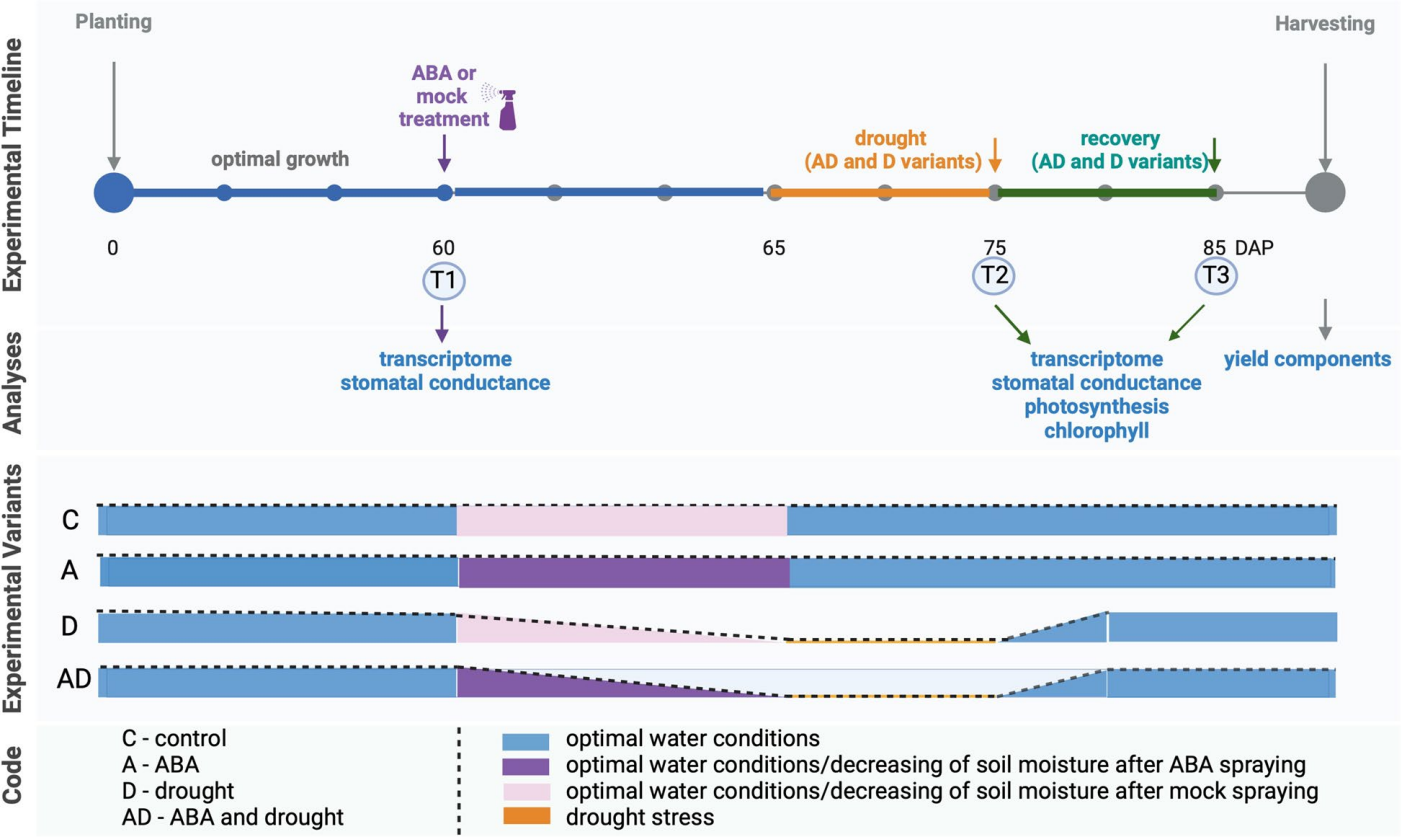
Experimental setup with conducted analyses at the indicated time points. Plants were treated with ABA on 60 DAP (Days After Planting, T1). Drought was applied on 65 DAP and lasted 10 days till 7P DAS (T2). The recovery period lasted 10 days till 85 DAP (T3). The indicated physiological analyses were performed for three biological replications, each replicate contained 5 plants. At the end of the experiment, post-harvest parameters were measured

We used barley plants (*Hordeum vulgare* (L.)) cultivar ‘Sebastian’, which is parental variety for TILLING population developed in our lab – *Hor*TILLUS [66]. Treatments included: untreated control ('C'), ABA application alone ('A'), ABA application followed by drought stress ('AD'), and drought stress alone ('D') (Fig. 1. Analyses were done at the end of the drought period (75 days,'T2'and after recovery (85 days,'T3'. Seeds germinated in Petri dishes were transferred to a soil mix in 13 cm × 13 cm × 13 cm pots. Growth was maintained under optimal water conditions (15% volumetric water content (vwc until 60DAP (Days After Planting (DAP. Starting at 60 DAP, we divided the plants into two groups for treatment: one received a 50 µM ABA spray ('A'and the other water ('C'. This led to four treatment designs mentioned above: Optimal water ('C'; ABA-treated under optimal water ('A'; ABA-treated, then drought from 65 DAP- 75 DAP ('AD'and Drought from 65 DAP- 75 DAP ('D'. The drought began at 65 DAP (heading stage: Z5.0; Zadok scale. The soil moisture was monitored daily using the TDR EasyTest (Institute of Agrophysics, Polish Academy of Sciences, Poland. In the Control treatment, the soil moisture remained at 15% vwc. The drought lasted for 10 d (soil moisture remained at 1.5–3% volumetric water content (vwc, after which moisture was restored to 15% vwc until 85DAP in the recovery phase. Growth was maintained in a controlled greenhouse until harvest. The experimental details are shown in Fig. 1. Leaf samples were collected at T1, T2, and T3 for transcriptomic analyses. Physiological analyses were performed under post-drought (T2) and post-recovery (T3) conditions.

### Physiological analysis

#### Chlorophyll a fluorescence

We measured chlorophyll *a* fluorescence [62], [15] with a Pocket PEA fluorimeter (Hansatech Instruments Ltd., England), dark-adapted third leaves for 30 min before exposing them to a light pulse (3500 µmol m^−2^ s^−1^) at 75 DAP post-drought and 85 DAP post-recovery. Data were analyzed using the Pocket PEA software (https://www.hansatech-instruments.com) followed by Biolyzer software (https://www.fluoromatics.com/biolyzer_software-1.php) and MS Office Excel with custom-made calculation sheet based on formulas provided in [15], with measurements taken from five plants per treatment, with three repetitions.

#### Chlorophyll content measurement

Chlorophyll was quantified using Dualex Scientific Plus (ForceA, France). Five plants from each treatment group were tested, with three replicates per treatment. Raw data was exported to MS Office Excel and further calculated.

#### Stomatal conductance

Stomatal conductance on the abaxial side of the third leaf was assessed using an AP4 porometer (Delta-T Devices, UK), which measures humidity differences between the environment and leaf (mmol m^−2^ s^−1^). Five plants from each treatment group were used in triplicate.

### Postharvesting analysis

After harvest, agronomic traits such as plant height, spike and awn length, number of spikes (both fertile and sterile), seed count, and weight of 1000 seeds were measured for 30 plants per treatment. Seeds were scanned with an EPSON PERFECTION V700 PHOTO scanner and analyzed using the Grain Scan software for attributes such as area, circuit, length, width, and coloration (Ch1, Ch2, and Ch3). In our experiment plants were treated with 50 µM ABA at booting stage (Z4.0; Zadok scale). To compare the effect of different ABA doses applied at different developmental stages on barley yield, we also sprayed plants with 25 µM ABA at tillering stage (Z2.0; Zadok scale), 25 µM ABA at booting stage and 50 µM ABA at tillering stage. Next, we applied drought stress to all ABA-treated plants as described in ‘Experimental setup’. In parallel, we cultivated control and only drought-treated plants. After plants reached full maturity, we conducted post-harvest analysis as described above.

### RNA extraction and transcriptome sequencing

For RNA-seq analyses, plant tissue (2 cm long fragments of the third leaf located 3 cm below the leaf tip) was collected in three biological replicates, each containing fragments from three independent seedlings. Total RNA from cv. ‘Sebastian’ leaf tissue was extracted using the miRvana isolation kit (ThermoFisher Scientific, USA) per manufacturer guidelines. The concentration and quality were assessed using a NanoDrop (ND- 1000) spectrophotometer, and the RNA integrity was verified using an Agilent 2100 Bioanalyzer with an RNA 6000 Nanochip. cDNA libraries prepared using Illumina TruSeq procedures were sequenced on an Illumina Novaseq 6000 sequencer at Macrogen: 2 × 150 bp PE reads, six samples per lane, and a full flow cell.

### RNA-seq analysis

Clean reads were mapped to the barley reference transcriptome (BaRTv2.18 [13]) using Kallisto v. 0.43.0 [5]. Differential gene expression analysis was conducted using 3DRNAseq in R environment [22]. The read counts and TPMs were generated using the tximport R package and Kallisto output, respectively. Low-expression transcripts and genes were filtered out based on the mean–variance trend. Genes were considered to be expressed if specific criteria on counts per million reads (CPM) were met. Normalization of read counts was performed using the TMM method, and PCA indicated no distinct batch effects. The Limma R package [53] was used to compare the expression levels. In all contrast groups, we analyzed the following: differentially expressed genes (DEG), which show significant expression changes between tested conditions; differentially expressed transcripts (DET), which are specific transcripts of genes exhibiting significant expression changes compared to other transcripts within the same gene; differential alternative splicing (DAS), where genes with multiple transcript isoforms display varying abundance patterns between conditions; and differential transcript usage (DTU), highlighting specific transcript variants preferentially expressed between tested conditions. The criteria for significant differential gene and transcript expression were set at log_2_FC ≥ 1 or ≤ − 1, with a Benjamini-Hochberg (BH) adjusted p-value < 0.01. To determine significant DAS, the percentage spliced (ΔPS) threshold was set to 0.5. DAS genes were identified in each control group based on an adjusted p-value below 0.01 and the presence of at least one transcript with a ΔPS greater than 0.5. For DTU identification, the expression of each transcript was compared to the weighted average expression of all other transcripts within the same gene, with a transcript classified as DTU if its adjusted p-value was < 0.01, and the absolute value of ΔPS exceeded 0.5. TopGO was used for Gene Ontology (GO) enrichment analysis, with visualization performed using the ggplot R package. Data are available at link refs. for the deposited database.

### Quantitative real-time PCR analysis

The expression of selected genes was analyzed using qRT-PCR. One microgram of total RNA underwent cDNA synthesized using the Maxima First Strand cDNA Synthesis Kit (Thermo Fisher Scientific). The diluted cDNA served as the qPCR template. Primers for qPCR were crafted using Primer3 software. The qPCR mix included cDNA, primer pair mixture, and 2 × Master Mix (Roche) and was run on a LightCycler 480 (Roche) using the SYBR Green I method. The protocol involved an initial denaturation, 45 cycles of temperature variation, and melting curve analysis. The reference genes were *EF1* (*elongation factor 1-a*) and HORVU6Hr1G085370 [32]. Data analysis was performed using LinRegPCR and Excel, with fold change calculated using the formula FC = E − ∆Ct, where E is the mean value of the amplification efficiency of a given gene and ∆Ct corresponds to the difference between the mean Ct values of all biological replicates between the two samples that were compared: A vs. C, D vs. C, or AD vs. C.

## Results

### ABA pretreatment helps in maintaining chlorophyll content and improves photosynthesis under drought

**Fig. 2:**
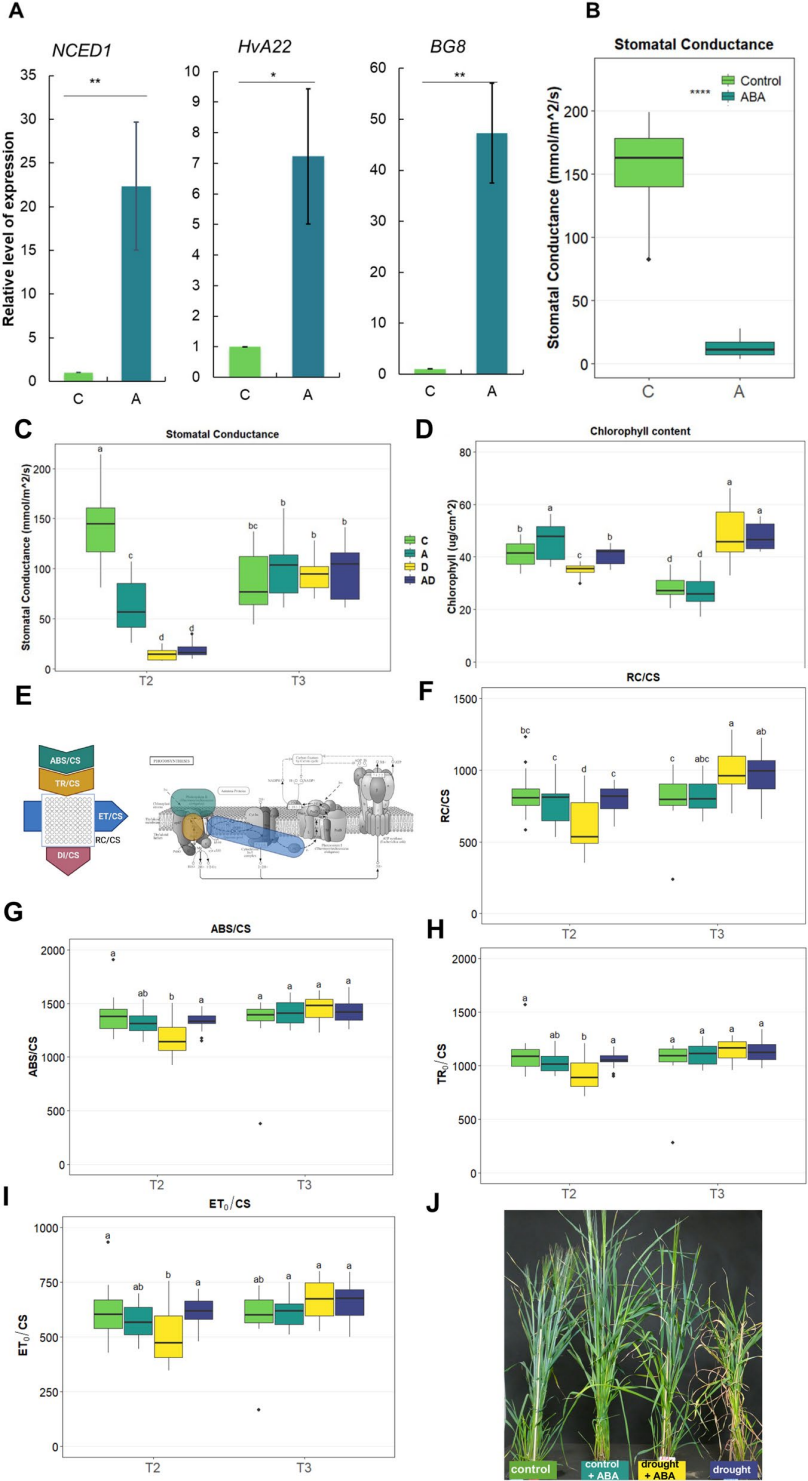
Physiological Parameters in Response to Treatments Applied. **A** Expression levels of *CAROTENOID CLEAVAGE DIOXYGENASE1* (*HvNCED1*), *HvA22*, and *β-GLUCOSIDASE8* (*HvBG8*) in response to ABA treatment at T1. Stars indicate statistically significant differences between groups: *P ≤ 0.05, **P ≤ 0.01, ***P ≤ 0.001, ****P ≤ 0.0001. **B** Stomatal conductance measured at T1, with significant differences between variants marked by stars as indicated above. Physiological parameters measured at T2 and T3: (**C**) Stomatal conductance at T2 and T3, (**D**) Chlorophyll content at T2 and T3, (**E**) Schematic representation of chlorophyll *a* fluorescence parameter measured and their role in the photosynthesis process. (**F-I**) Photosynthetic efficiency parameters: (**F**) Active PSII reaction centers per cross-section (RC/CS), (**G**) Absorption flux per cross-section (ABS/CS), (**H**) Trapping flux per cross-section (TR0/CS), and (**I**) Electron-transport flux per cross-section (ET0/CS). One-way ANOVA (*P* ≤ 0.05) followed by Tukey HSD test (*P* ≤ 0.05) was applied to identify significant differences between experimental variants. Statistically significant differences (*P* ≤ 0.05) are denoted by different letters, (**J**) Representative images of barley plants under control, control + ABA, drought + ABA, and drought conditions at T2

To investigate the role of ABA pretreatment in barley drought response using a carefully structured experimental design. We used four treatment variants: (i) optimal water conditions for the whole barley life cycle (C, control), (ii) ABA spray applied at 60 DAP (T1) at the booting stage, followed by growth in optimal water conditions (A, ABA); (iii) ABA spray applied at 60 DAP (T1), drought stress applied for 10 days 65–75 DAP (T2), and recovery phase until 85 DAP (T3) [AD, ABA + drought]; (iv) drought stress applied for 10 days 65 - 75DAP (T2), and recovery phase until 85 DAP (T3) [D, drought] (Fig. 1). To confirm the effects of ABA spraying, we measured the stomatal conductance and expression levels of selected ABA-responsive genes 3 h after foliar application. ABA triggered stomatal closure and upregulated the expression of the ABA biosynthesis gene *HvNCED1* (*9-cis-epoxycarotenoid dioxygenase*), the ABA-responsive *HvA22* gene, and the ABA conjugation gene *HvBG8* (*ABA-GE esters by beta-glucosidases*) (Fig. 2A). As stomatal closure is one of the earliest plant responses to drought stress, we measured stomatal conductance at T1, T2, and T3 across all experimental variants (Fig. 2B). Drought-treated plants (D) showed more pronounced stomatal closure than did ABA-treated plants (A). However, stomatal closure in drought-treated plants (D) was similar to that of plants pretreated with ABA and subjected to drought (AD). After the recovery phase (T3), stomatal conductance did not differ significantly among the variants (Fig. 2C). Drought stress reduces the chlorophyll content, which negatively affects photosynthesis. At T2, plants sprayed with ABA (A) accumulated significantly more chlorophyll than did the control plants. As expected, drought treatment (D) caused a significant decrease in the chlorophyll content compared to that of the control. In contrast, plants pretreated with ABA and exposed to drought (AD) retained a significantly higher chlorophyll content than plants treated with drought alone (D). These findings indicate that ABA pre-treatment preserves chlorophyll biosynthesis and promotes stability under drought conditions. Surprisingly, after the recovery phase (T3), drought-treated plants (D) and ABA + drought plants (AD) exhibited higher chlorophyll contents than the control (C) (Fig. 2D).

Photosynthetic performance typically decreases under drought stress owing to stomatal closure and increased reactive oxygen species (ROS) activity. Efficient photosynthetic protection plays a crucial role in plant adaptation to drought. To determine whether ABA pre-treatment preserves photosynthetic performance under drought conditions, we measured photosystem II (PSII) activity using chlorophyll a fluorescence [27], [51]. The analysis focused on biophysical parameters, including the absorption flux per cross-section (ABS/CS), trapping flux (TR_0_/CS), and electron transport flux (ET_0_/CS), which describe the absorbed energy, trapped energy, and transferred electrons in PSII, respectively. We also assessed the number of active reaction centers in PSII (RC/CS) to evaluate the electron flow within the photosynthetic machinery (Fig. 2E). Under drought stress, the RC/CS, ABS/CS, TR_0_/CS, and ET_0_/CS decreased significantly. However, ABA-treated (A) and ABA + drought-treated plants (AD) showed no significant differences compared to the control at T2 (Fig. 2F-I). Notably, plants subjected to drought (D) and ABA + drought (AD) displayed an increased number of reaction centers (RC) after the recovery phase (T3) (Fig. 2F). These results demonstrate that ABA pre-treatment stimulates protective mechanisms in photosynthesis, allowing plants to maintain efficient photosynthetic performance under drought conditions. Moreover, plants exposed to drought phenotypically exhibited pronounced wilting, leaf curling, and chlorosis, indicative of impaired physiological status compared to control conditions. In contrast, ABA-pretreated plants displayed reduced symptoms of drought stress, maintaining improved turgor, greener foliage, and minimal leaf damage (Fig. 2J). These observations suggest that ABA pretreatment effectively mitigates drought-induced physiological impairment, supporting sustained photosynthetic efficiency under water deficit conditions.

### ABA pretreatment activated earlier stress pathways in drought-stressed plants based on transcriptome reprogramming

**Fig. 3:**
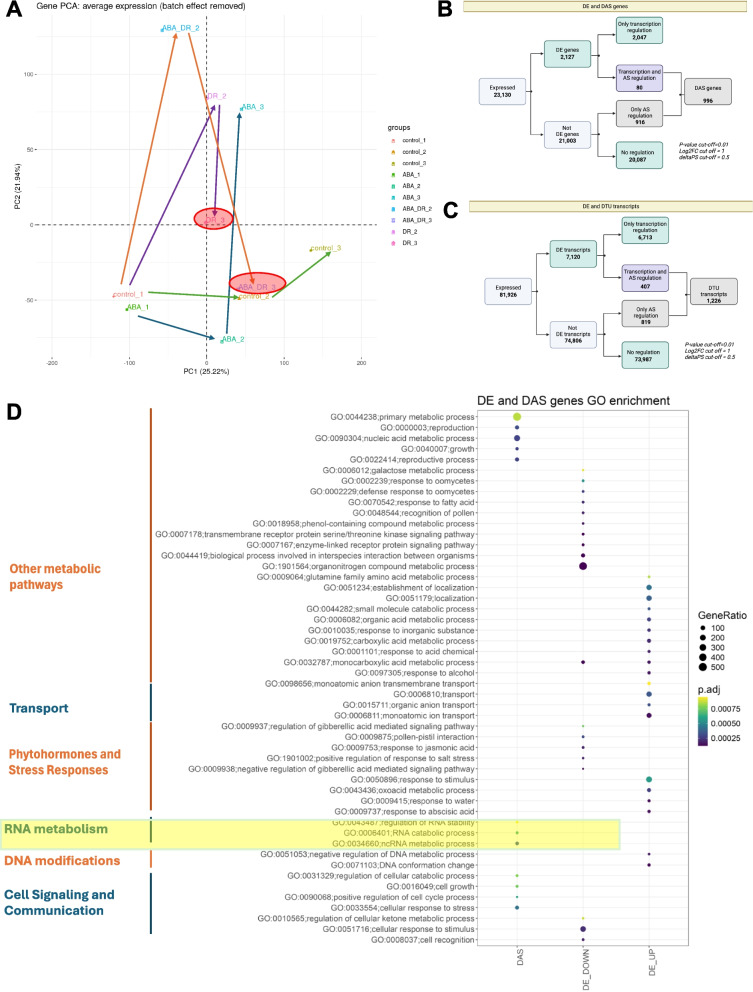
Transcriptome response to drought and drought when pretreated with ABA (**A**) PCA analysis (**B**) Differential Expression Analysis at the gene-level; (**C**) Differential Expression Analysis at the transcript-level; (**D**) Gene Ontology Enrichment analysis of Differentially Expressed Genes (DEGs) and Differential Alternatively Spliced Genes (DAS)

To determine whether ABA pretreatment enables the earlier activation of stress pathways, we conducted a comprehensive comparative RNA-seq analysis of ABA-pretreated plants subjected to drought stress (AD) and drought-only treated plants (D) after stress exposure (T2) and the rewatering phase (T3). Principal Component Analysis (PCA) showed that plants pretreated with ABA and exposed to drought (AD2) grouped differently from those subjected solely to drought. Notably, plants pretreated with ABA during the recovery phase (AD3) grouped closely with the control plants (C2), unlike drought-only plants, which did not return to similar recovery positions (Fig. 3A).

We further examined changes in gene expression at both the gene and transcript levels (Supplementary Table 1). Out of 2,127 differentially expressed genes (DEGs), 80 were regulated by both transcriptional activity and alternative splicing in response to drought. Additionally, 916 genes that were not identified as differentially expressed were exclusively regulated by splicing (Fig. 3B). At the transcript level, 819 transcripts that were not classified as differentially expressed transcripts (DETs) underwent regulation via alternative splicing (Fig. 3C). These observations suggest that splicing significantly drives the observed expression changes and likely plays a key role in mediating plant responses to ABA pre-treatment and drought stress. To explore the biological significance of DEGs and differentially alternatively spliced genes (DAS), we performed Gene Ontology (GO) enrichment analysis on all upregulated and downregulated DEGs, as well as on all DAS genes (Fig. 3D). This analysis revealed that only DAS genes were significantly enriched in processes related to RNA metabolism, highlighting the central role of splicing in RNA regulation under stress conditions. Furthermore, DAS genes were predominantly associated with growth and reproductive processes, which were particularly critical given the timing of drought application in this study. Interestingly, upregulated DEGs were associated with responses to abscisic acid, water stress, other environmental stimuli, and DNA modification. The enrichment of DNA modification-related processes is particularly noteworthy, as subsequent studies have provided further insights into the mechanisms underlying ABA action.

### Differential gene and transcript expression dynamics under drought and drought following ABA pretreatment

**Fig. 4:**
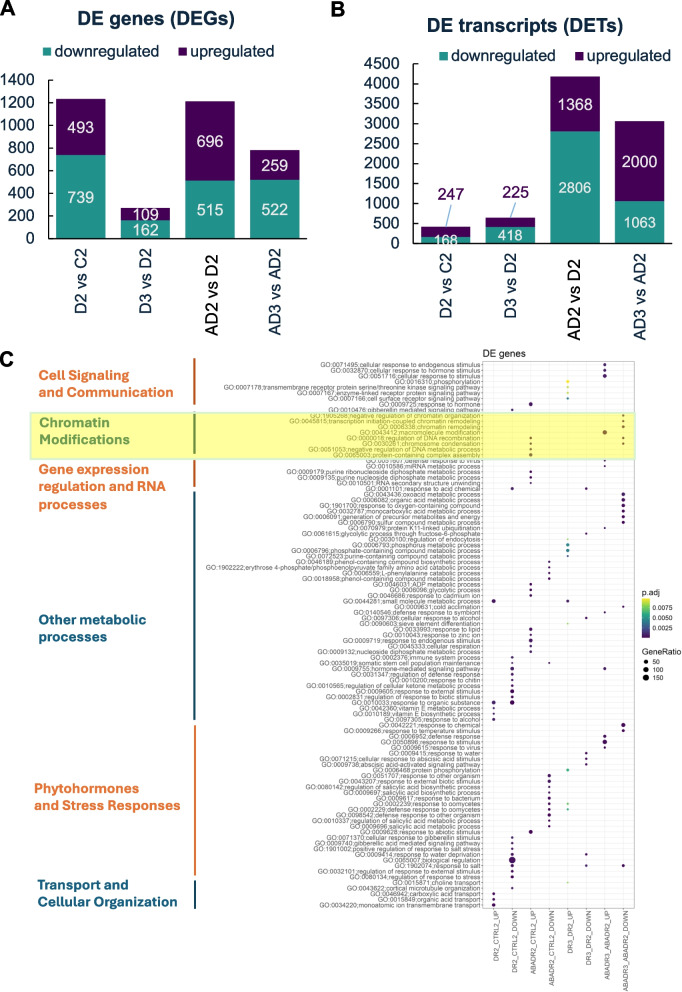
Analysis of Differentially Expressed Genes (DEGs) (**A**) and Differentially Expressed Transcripts (DETs) (**B**) number in the studied contrast groups. **(C)** Overrepresented biological processes (BP) identified for DEGs

We conducted pairwise differential gene expression analyses between experimental variants at consecutive time points at both gene (differentially expressed genes, DEGs) and transcript (differentially expressed transcripts, DETs) levels. Drought treatment induced a higher number of DEGs (1,232), but significantly fewer DETs (415) (Fig. 4AB). In contrast, ABA-pretreated plants subjected to drought (AD2) exhibited a comparable number of DEGs (1,211) but a significantly higher number of DETs (4,174) than plants treated solely with drought (D2). The most striking differences were observed in the recovery phase. ABA-pretreated plants after recovery showed a substantially higher number of DEGs (AD3:781) than drought-only treated plants at the same time-point (D3:271) (Fig. 4AB). Similarly, the number of DETs increased in both recovery groups, but plants recovering after ABA pre-treatment followed by drought (AD3) displayed markedly higher transcript-level changes (3,063) than drought-only plants (D3:643). These results emphasize the robust influence of ABA pre-treatment on differential gene and transcript expression dynamics during stress recovery (Fig. 4B). GO enrichment analysis of DEGs revealed that genes related to DNA, particularly chromatin modifications, were predominantly upregulated in ABA-pretreated plants subjected to drought. These included processes, such as chromosome condensation (GO:0030261). During the recovery phase, downregulated genes in the ABA-pretreated variant exhibited even greater enrichment in chromatin and DNA modification processes, including chromatin remodeling (GO:0006338), transcription initiation-coupled chromatin remodeling (GO:0045815), and negative regulation of chromatin organization (GO:1,905,268). A similar pattern was observed for genes associated with RNA metabolism, indicating a consistent regulatory trend across the treatment and recovery stages (Fig. 4C). These findings suggest dynamic adjustments in gene expression related to chromatin and RNA metabolism, which depend on both the treatment conditions and the recovery phase.

### Enhanced alternative splicing regulation in ABA pretreated plants under drought and recovery conditions

**Fig. 5:**
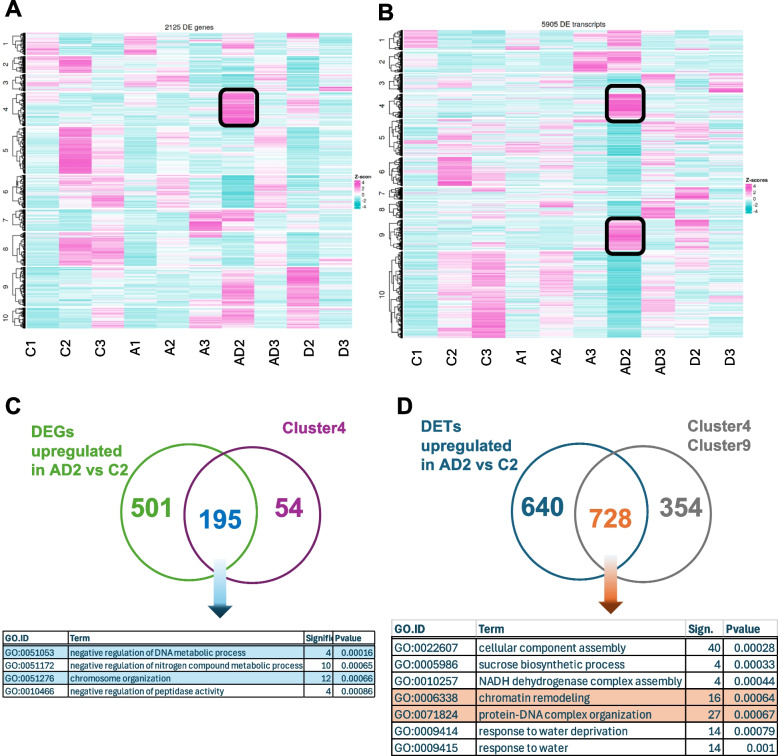
Hierarchical clustering of DEGs (**A**) and DETs (**B**); Overrepresented biological processes (BP) identified for DEGs classified in Cluster 4 that overlaps with upregulated DEGs in AD2 variant (**C**); Overrepresented biological processes (BP) identified for DETs classified in Cluster 4 and 9 that overlaps with upregulated DETs in AD2 variant (**C**)

We investigated whether splicing events were specific to the treatments, as suggested by the earlier results (Table 1). To explore this, we compared the number of differentially expressed genes (DEGs) and differentially alternatively spliced genes (DAS) across two conditions: plants pretreated with ABA followed by drought (AD), and plants subjected to drought alone (D). We analyzed the outcomes during both the drought and recovery phases (Table 1). Plants treated only with drought exhibited a higher number of DAS during recovery than during the drought phase. However, ABA-pretreated plants showed significantly higher splicing activity during both the drought and recovery phases, as evidenced by the elevated number of DAS. This highlights the pronounced role of alternative splicing in ABA-pretreated plants, enhancing their ability to adapt to drought and recovery conditions. Based on these findings, we conducted a deeper investigation of the regulation of transcript-level expression. Since the drought phase (T2) is crucial for understanding ABA-induced differences in stress responses, we speculated that genes responsible for earlier ABA signaling activation might display distinctive expression patterns at T2, followed by changes during recovery (T3). To test this, we performed hierarchical clustering of all DEGs and DETs (Fig. 5AB). For DEGs, Cluster 4 demonstrated high peak of expression at AD2, prompting further analysis. Overlap analysis between Cluster 4 and DEGs upregulated in the AD2 variant identified 195 common genes enriched in processes, such as negative regulation of DNA metabolic processes (GO:0051053; 4 genes) and chromosome organization (GO:0051276; 10 genes). Among these processes, BaRT2v18chr5HG271230, which encodes an H15 domain-containing DNA-binding protein, exhibited the highest expression (log_2_FC = 3.71) (Fig. 5C). Next, we focused on Clusters 4 and 9 of differentially expressed transcripts (DETs) in ABA-pretreated plants exposed to drought (Fig. 5D). These clusters showed a unique transcriptional upregulation. Overlap analysis between genes grouped in C4 and C9, with DETs upregulated in the AD2 variant, revealed 728 DETs. The GO enrichment analysis highlighted the biological relevance of these transcripts (Fig. 5D). In Cluster 4, the most significant biological processes (BPs) were related to chromatin modifications, a feature absent in drought-only plants (Supplementary Table 2). Notably, transcripts in C4 remained unchanged under other conditions, indicating that this response is unique to ABA-pretreated plants in response to drought.

**Table 1:** Comparison of Differentially Expressed Genes (DEGs) and Differential Alternatively Spliced Genes (DAS) Across Various Experimental Conditions

**contrast group**	**DEG**	**DAS**	**DEG+DAS**
D2 vs C2	1231	39	1
AD2 vs C2	1187	495	24
D3 vs D2	270	117	1
AD3 vs D2	771	267	10

In ABA-pretreated plants exposed to drought, upregulation of specific transcripts in C4 underscores a sophisticated adaptive mechanism that enhances plant resilience. For instance, the transcript encoding phosphoethanolamine N-methyltransferase (BaRT2v18chr1HG039630) plays a critical role in modifying membrane lipids that maintain cellular structure and function under stress. Chromatin remodeling proteins, such as CHROMATIN REMODELING 5 (BaRT2v18chr2HG061080) and components of the chromatin-remodeling complex ATPase (BaRT2v18chr3HG120910), regulate gene expression by restructuring chromatin. Additionally, transcripts for adenosylhomocysteinase (BaRT2v18chr2HG104790 and BaRT2v18chr2HG105160) support the methylation cycle and influence metabolite synthesis and gene regulation. The upregulation of Evolutionarily Conserved C-terminal Region 7 (BaRT2v18chr3HG137030) ensures efficient RNA processing and protein synthesis, whereas H15 domain-containing proteins (BaRT2v18chr5HG271230, BaRT2v18chr5HG271250, and BaRT2v18chr5HG271320) and DNA replication factors (BaRT2v18chr6HG304230) contribute to DNA integrity, replication, and repair processes (Supplementary Table 2). These genetic responses collectively stabilize cellular processes, regulate gene expression, and adjust metabolic pathways, thereby enhancing the ability of plants to withstand and recover from drought stress.

In contrast, cluster 9 revealed significant enrichment in BPs associated with responses to abiotic stimuli, abscisic acid, and monoatomic ion transmembrane transport. Although this cluster was also upregulated in drought-only plants, the changes were more pronounced in ABA-pretreated plants. This suggests that ABA pre-treatment amplifies drought responses through specific transcriptional and post-transcriptional modifications. Transcripts in these processes included ABA-responsive markers, such as the dehydration-responsive protein RD22-like, SnRK2 kinases, phosphatases, and ABA-responsive transcription factors, which indicate a strong ABA-mediated response (Supplementary Table 2)*.* In ABA-pretreated plants subjected to drought stress, upregulation of specific genes further supports an orchestrated ABA response. Transcripts encoding chlorophyll a-b-binding proteins and chloroplast-specific enzymes maintain photosynthetic efficiency and protect cellular structures. Genes associated with amino acid metabolism, lipid metabolism, and energy production underscored the recalibration of the metabolic pathways to endure drought stress. These molecular adjustments collectively sustain cellular integrity and function under conditions of water scarcity.

Given the observed upregulation of specific transcripts, particularly in ABA-pretreated plants, alternative splicing has emerged as a pivotal mechanism in stress response. The pronounced increase in differentially alternatively spliced genes (DAS) highlights the role of alternative splicing in ABA-dependent adaptation to drought and recovery conditions.

### Role of isoform switching in ABA pretreated plants under drought stress and recovery

**Fig. 6:**
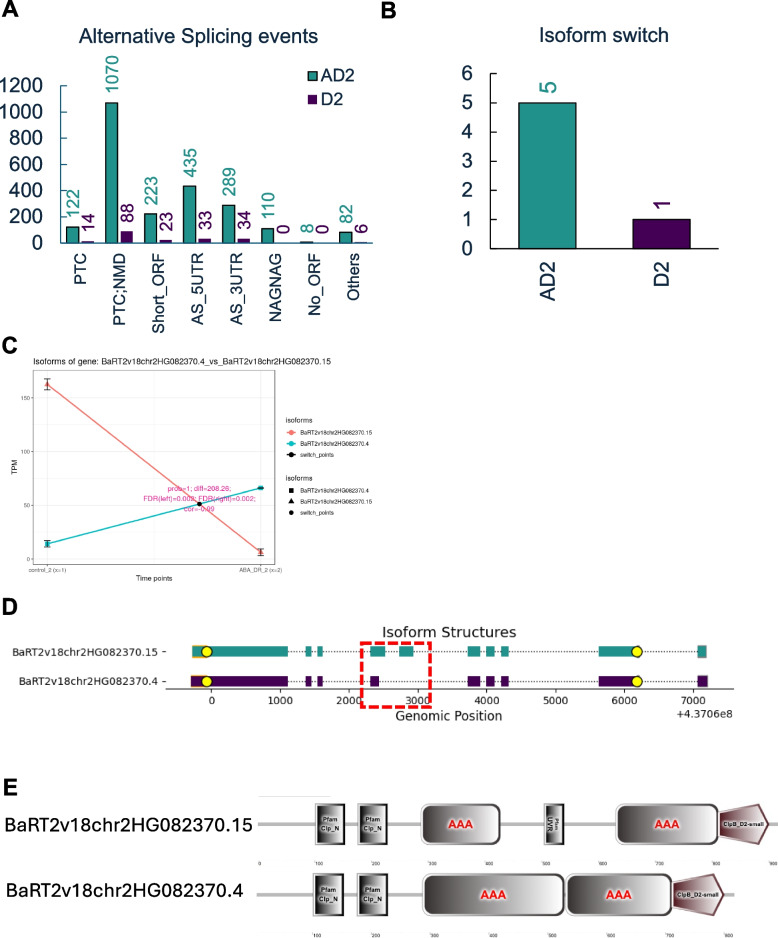
Alternative splicing (AS) regulation under drought and when barley plants were exposed to drought after ABA pretreatment. The number and type of AS events in AD2 and D2 variant (**A**); The number of Isoform switches identified in AD2 and D2 variant (**B**); Isoform switch between control and drought conditions in AD2 variant (**C**); Structure of each of two isoforms identified as isoform switch (**D**); Predicted domain structure of each of isoforms (**E**)

To further investigate the role of alternative splicing (AS) in plant responses to drought stress, we analyzed the types of AS events under two experimental conditions: drought-only treated plants and ABA-pretreated plants exposed to drought. In both cases, nearly 50% of differentially expressed transcripts (DETs) contained premature termination codons (PTCs), highlighting the significant regulatory role of nonsense-mediated decay (NMD). Drought-only treatment resulted in 213 identified splicing variants, whereas ABA pretreatment followed by drought stress led to a substantial increase, with 2,339 splicing variants identified (Fig. 6A). This striking difference underscores the critical role of splicing regulation in ABA-mediated responses, suggesting that ABA pretreatment amplifies a plant ability to modulate its transcriptome through alternative splicing, preparing it for subsequent drought stress.

The analysis of splicing events has led to the exploration of isoform switching, in which a single gene generates multiple mRNA isoforms that can produce proteins with distinct functions. Isoform switching can profoundly influence cellular behavior and enhance plant resilience to stress, providing an additional layer of regulation that is activated by ABA pretreatment. This highlights transcriptome plasticity under environmental stress and suggests potential strategies for enhancing stress tolerance by manipulating splicing patterns. In our study, ABA-pretreated plants displayed a significant increase in isoform switching (IS), with five IS identified, compared to only one IS in drought-only treated plants (Fig. 6B). These findings support the hypothesis that ABA pretreatment promotes a more dynamic and flexible transcriptomic response, particularly through alternative splicing and isoform switching, enhancing the capacity of plants to adapt to stress. A notable isoform switch occurred in BaRT2v18chr7HG383330, a gene identified as differentially alternatively spliced (DAS) and represented by differentially expressed transcripts (DET) in response to ABA pre-treatment followed by drought stress (AD) (Fig. 6C,D). After drought stress, the isoform BaRT2v18chr7HG383330.2 was downregulated (log_2_FC = − 1.48), but it was upregulated during the recovery phase (log_2_FC = 1.34). This gene encodes an XH domain-containing protein homologous to Arabidopsis IDN2 (INVOLVED IN DE NOVO 2), a key component of the RNA-directed DNA methylation (RdDM) pathway that regulates de novo methylation and siRNA-mediated maintenance methylation. A significant isoform switch occurred specifically in the ABA-treated drought variant (ABADR2), where BaRT2v18chr7HG383330.2 was downregulated, and a truncated isoform, BaRT2v18chr7HG383330.3, lacking part of the N-terminal functional domain, were upregulated in response to drought (Fig. 6CDE). This evidence highlights the adaptive significance of isoform switching in the response to environmental stress. The switch to a less functionally complex isoform under severe drought stress suggests strategic adaptation to reduce metabolic costs and sustain essential functions (Fig. 6F). As the RdDM pathway primarily regulates gene silencing and transcriptional regulation, this isoform switching implies that drought responses also involve long-term genetic modifications. These modifications can permanently alter gene expression patterns, enabling plants to"remember"and respond more efficiently to recurring stressful events.

### Plants pre-treated with ABA before drought showed differentiated expression of genes related to photosynthesis

Physiological analyses revealed that ABA-pretreated plants subjected to drought stress exhibited improved photosynthetic parameters. To investigate the molecular basis of this observation, we analyzed the differentially expressed genes (DEGs) associated with photosynthesis. Drought treatment alone specifically affected the expression of 32 genes, including 23 upregulated and 9 downregulated genes, all annotated with GO terms such as photosynthesis, photosystem, chlorophyll, and chloroplast. During the recovery phase, drought-only plants exhibited specific differential expression of 36 genes, with 31 upregulated and 5 downregulated. In ABA-pretreated plants subjected to drought (AD variant), 31 photosynthesis-related genes were found to be specifically upregulated, whereas 5 were specifically down-regulated (Supplementary Table S1).

Among the genes upregulated during drought in ABA-pretreated plants and subsequently downregulated after recovery were BaRT2v18chr2HG083660, encoding a DUF1279 domain-containing protein; BaRT2v18chr7HG353680, encoding glutamate- 1-semialdehyde 2,1-aminomutase; BaRT2v18chr1HG028690, encoding tocopherol cyclase; BaRT2v18chr1HG022840, encoding an ABC-type Co^2^⁺ transport system permease component; and BaRT2v18chr7HG379120, encoding ATP-dependent zinc metalloproteases. The DUF1279 domain-containing protein facilitates protein–protein interactions, which are critical for stress response mechanisms. Glutamate- 1-semialdehyde 2,1-aminomutase contributes to chlorophyll biosynthesis and directly enhances the photosynthetic efficiency. Tocopherol cyclase synthesizes tocopherols (vitamin E), which protect the chloroplast membranes from oxidative damage during stress. The ABC-type Co^2^⁺ transport system permease component regulates ion transport and homeostasis, thereby supporting cellular stress responses. ATP-dependent zinc metalloproteases, including FTSH proteins, play essential roles in protein quality control by degrading damaged proteins within chloroplasts, thereby maintaining cellular functions under stress conditions. Notably, these genes were also upregulated in drought-only plants; however, they remained unaffected during the recovery phase in this variant. This difference suggests that although these genes play crucial roles in the immediate drought response, their downregulation in ABA-pretreated plants during recovery indicates a shift from the stress response to the recovery and repair processes. ABA pre-treatment likely accelerates this shift, enabling plants to restore normal cellular functions. The observed downregulation of these genes during re-watering in ABA-pretreated plants reflects the plant's need to reduce defensive activity and prioritize resource conservation under restored conditions. This dynamic regulation ensures that enzymes critical for the stress response remain active during drought but are minimized during recovery, thereby optimizing cellular homeostasis and resource allocation. Our findings demonstrated that ABA pretreatment primes plants for enhanced drought tolerance by upregulating photosynthesis-related genes during stress. The subsequent downregulation of these genes upon re-watering highlights an efficient transition from defense to recovery, promotes resource conservation, and restores normal cellular processes.

### ABA application stimulates the ABA pathway in response to drought

**Fig. 7:**
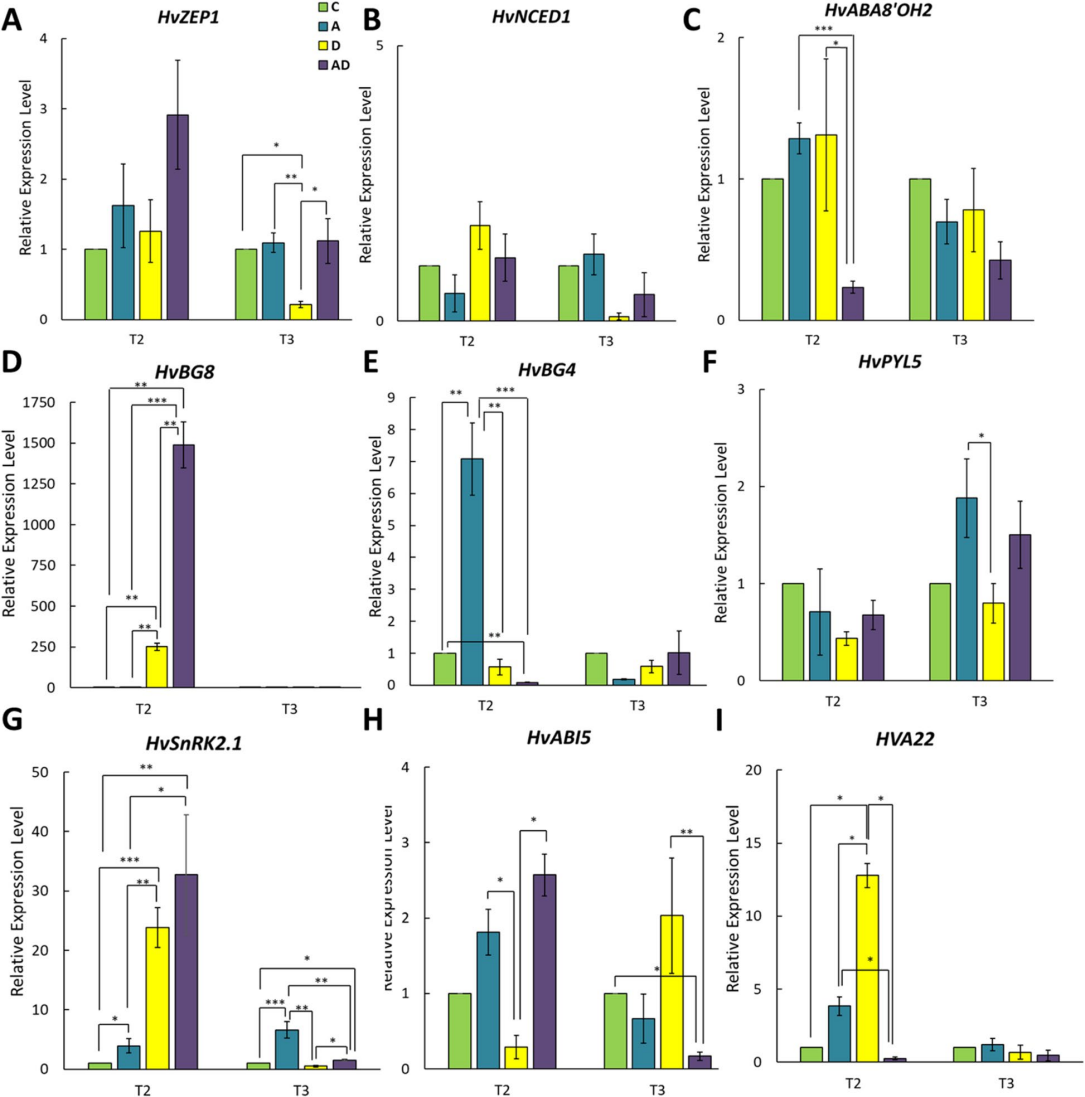
Expression analysis of genes involved in ABA pathway in control (**C**) plants and exposed to ABA (**A**), drought (**D**), and ABA pretreatment followed by drought (AD) at T2 and T3. (**A**) *ZEAXANTHIN EPOXIDASE1*—*HvZEP1*, (**B**) *CAROTENOID CLEAVAGE DIOXYGENASE1*—*HvNCED1*, **(C)** *ABA8’-HYDROXYLASE2*—*HvABA8’OH2*, (**D**) *β-GLUSIDASE8*—*HvBG8*, (**E**) *HvBG4*, (**F**) *PYRABACTIN RESISTANCE 1-LIKE 5*—*HvPYL5*, (**G**) *SNF1-RELATED PROTEIN KINASE 2.1*—*HvSnRK2.1*, (**H**) *ABA INSENSITIVE5*—*HvABI5* and (I) *HvA22*. T-test was applied to identify differences between analyzed variants of the experiment. Statistically significant differences between indicated groups are marked by stars—^*^P ≤ 0.05, ^**^P ≤ 0.01, ^***^P ≤ 0.001

Physiological assays revealed that ABA pre-treatment at the booting stage improved photosynthetic efficiency in barley subjected to drought stress during the heading stage. We hypothesized that exogenous ABA stimulates the ABA pathway, thereby enhancing the ability of plants to respond to drought. However, global transcriptome analysis did not reveal significant changes in the expression of essential ABA pathway-related genes in the ABA-pretreated drought variant (AD). To verify this, we analyzed the expression of ABA metabolism and signaling genes under drought stress (T2; 75 DAP) and during the recovery phase (T3; 85 DAP) using RNA-seq data (TPM values, Supplementary Table S3) and RT-qPCR. In the AD variant, ABA-pretreated plants exhibited higher TPM values for *HvBG8*, whereas *HvABAOH2* and *HvBG4* showed lower values at T2 than the other variants. RT-qPCR data confirmed these observations. Additionally, *HVA1* and *HVA22* displayed higher TPM values in the AD variant during T2. RT-qPCR analysis was focused on specific ABA-related genes. *HvZEP1* and *HvNCED1* are key genes involved in ABA biosynthesis and regulate critical steps in ABA production. HvZEP1 converts zeaxanthin to violaxanthin, whereas HvNCED1 catalyzes the cleavage of 9-cis-violaxanthin and 9-cis-neoxanthin into xanthoxin [57]. It should be emphasized that *HvZEP1* could also be involved in the xanthophyll cycle as its ortholog in Arabidopsis [47, 55]. Notably, *HvZEP1* showed no significant changes at T2; however, its expression decreased in drought-only plants (D) during recovery at T3 (Fig. 7A). In contrast, *HvNCED1* exhibited no significant difference in expression throughout the experiment (Fig. 7B). For ABA catabolism, we analyzed *HvABA8’OH2*, a gene encoding ABA 8'-hydroxylase, which degrades ABA to ABA8'OH [33]. In barley, three genes are involved in ABA catabolism (*HvABA8’OH1 - 3*) [57]. At T2, *HvABA8’OH2* showed reduced expression in the AD variant compared to that in the drought-only (D) plants (Fig. 7C). No significant differences were observed at the other time points.

ABA levels are also regulated through the conjugation and deconjugation of ABA-glucose esters (ABA-GE), which involve beta-glucosidases (BGs) [24]. In barley, *HvBG8* and *HvBG4* control ABA deconjugation and conjugation [56], [39]. At T2, *HvBG8* expression increased in both D and AD variants, with nearly sixfold higher expression in the AD variant (Fig. 7D). After recovery to T3, *HvBG8* expression returned to baseline levels. For *HvBG4*, expression decreased significantly in the AD variant at T2 compared to the control plants (C), whereas no changes occurred in drought-only plants (D) (Fig. 7E). We also examined *HvPYL5*, an ABA receptor, and *HvSnRK2.1*, a key ABA-dependent kinase [56]. *HvPYL5* levels showed no significant changes at T2 or T3 (Fig. 7F). In contrast, *HvSnRK2.1* exhibited upregulated expression in both D and AD variants at T2 compared to control plants. However, expression remained higher in the AD variant at T3, whereas drought-only plants showed reduced HvSnRK2.1 expression after recovery (Fig. 7G). *HvABI5*, an ABA-dependent transcription factor, regulates stress-responsive genes such as *HVA1* and *HVA22*. At T2, *HvABI5* activity decreased in drought-only plants (D), but remained elevated in ABA-pretreated plants (AD). By T3, *HvABI5* expression was significantly decreased in the AD variant compared to the control (C) and drought-only plants (D) (Fig. 7H). *HVA22*, a target of *HvABI5,* encodes LEA protein involved in vesicular transport. Our previous studies revealed that *HVA22* is induced by drought applied at seedling stage in barley [8, 12]. HVA22 was significantly upregulated at T2 in drought-only plants compared to controls. However, *HVA22* expression was lower in ABA-pretreated plants (AD) at T2, with no significant changes observed during recovery (T3) (Fig. 7I).

These results suggest that ABA pretreatment enhances drought response by upregulating critical genes within the ABA signaling pathway. Dynamic regulation of these genes highlights the role of hormonal priming in enhancing drought tolerance. ABA pretreatment promotes robust gene activation during drought stress while facilitating a timely transition to recovery processes.

### Timing and concentration of ABA pretreatment significantly impacts yield parameters in barley under drought stress

**Fig. 8:**
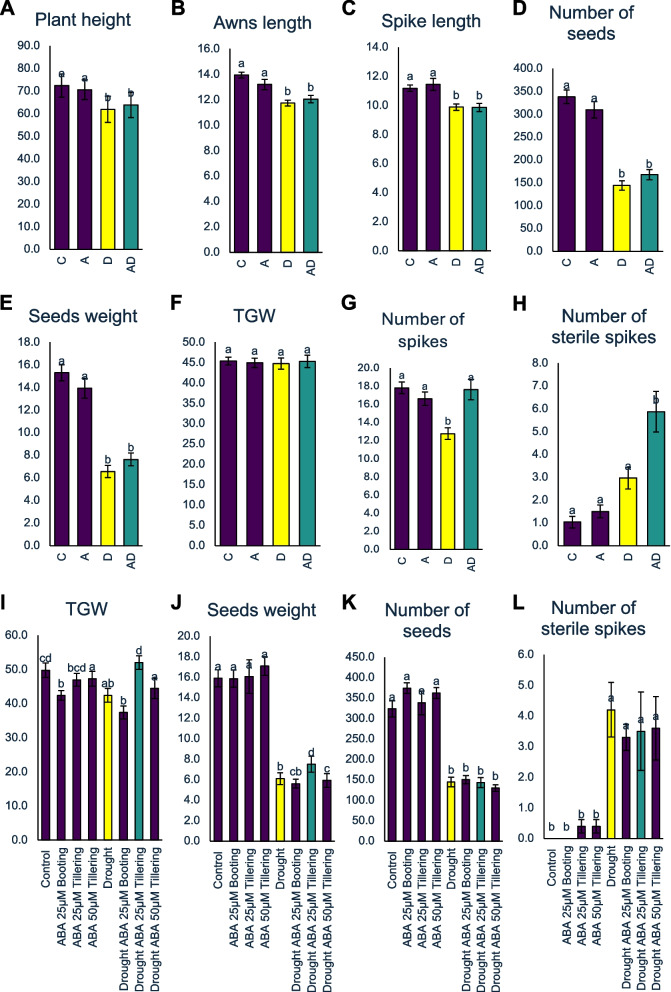
Post-harvest analysis (**A**) Plant height, (**B**) awns length, (**C**) spike length, (**D**) number of seeds, (**E**) seed weight per plant, (**F**) thousand grain weight (TGW), (**G**) spikes number, (**H**) sterile spikes number. Post-harvest analysis of the second experiment with dose- and time-changes: (**I**) thousand grain weight (TGW), (**J**) seed weight per plant, (**K**) number of seeds, (**L**) sterile spikes number. One-way ANOVA (P ≤ 0.05) followed by Tukey HSD test (*P* ≤ 0.05) was applied to identify differences between analyzed variants of the experiment. Statistically significant differences (*P* ≤ 0.05) are indicated by different letters

To evaluate the effect of ABA pretreatment on barley growth and yield under drought conditions, we conducted a comprehensive post-harvest analysis of all experimental variants. We measured key yield components, including plant height, spike and awn lengths, seed number and weight per plant, thousand grain weight (TGW), and number of spikes and sterile spikes. Drought (D) and ABA-pretreated plants subjected to drought (AD) showed significant reductions in plant height, spike length, awn length, seed number, and seed weight compared with the control. In contrast, ABA treatment alone (A) had no effect on these parameters (Fig. 8A–E). Notably, TGW remained unaffected across all the treatments (Fig. 8F). The total number of spikes per plant decreased significantly under drought stress. However, plants in the ABA and AD variants maintained a comparable number of spikes as the control plants (Fig. 8G). Interestingly, the combination of ABA pre-treatment and drought stress (AD) led to a higher number of sterile spikes per plant compared to the other experimental variants (Fig. 8H), suggesting a possible negative interaction between ABA pre-treatment and drought stress in this context. To gain deeper insights into seed parameters, we conducted an image-based analysis using the GrainScan software [71]; Supplementary Fig. 4). The analysis included measurements of the seed area, circuit, length, width, and pigmentation (Ch1, Ch2, and Ch3 parameters). Both drought (D) and AD treatments significantly reduced the seed circuit, seed length, and pigmentation parameters, whereas ABA treatment alone showed no significant changes compared to the control (Supplementary Fig. 4). Importantly, the seed area values in the ABA and AD variants remained similar to those of the control plants, whereas drought alone caused a significant reduction in this parameter. Seed width did not differ across any of the analyzed variants (Supplementary Fig. 4). The observed increase in sterile spikes following ABA pre-treatment, combined with its beneficial effects on photosynthesis, prompted further investigation to determine whether the negative effects on spike fertility could be mitigated. We designed a follow-up experiment using adjusted ABA concentrations and altered the timing of the application. Specifically, barley plants were treated with 50 µM ABA during the tillering stage and with a reduced dose of 25 µM ABA at the booting stage. In addition, a separate group received 25 µM ABA earlier during the tillering stage. We measured photosynthetic parameters after drought exposure (75 DAP) and obtained results consistent with those of the earlier experiment, confirming improved photosynthetic efficiency under ABA pretreatment. Next, we compared the yield components under modified conditions. Notably, earlier ABA application at a lower concentration (25 µM during the tillering stage) resulted in significantly higher thousand grain weight (TGW) and seed weight per plant compared with plants treated solely with drought. Importantly, the number of sterile spikes in this treatment group was comparable to that observed in the drought-only plants (Fig. 8I–L). These findings demonstrate that the beneficial effects of ABA pretreatment on photosynthesis and yield parameters depend on the timing and concentration of ABA application. Although direct ABA application may be impractical for breeders because of its high cost, the observed mechanisms provide valuable insights for alternative approaches. For example, breeding programs can focus on selecting or developing barley varieties with enhanced endogenous ABA responses, earlier activation of stress pathways, or improved alternative splicing efficiency. Additionally, hormone analogs, biostimulants, and agronomic practices that mimic ABA's effects of ABA on photosynthesis and drought recovery could offer cost-effective solutions to improve yield stability under water-limited conditions.

## Discussion

Adverse effects of drought stress on plant growth, development, and productivity have long been recognized. In the present study, we deepened our understanding of the role of abscisic acid (ABA) in improving drought tolerance in barley by focusing on its effects on transcriptional reprogramming including alternative splicing events. Our study demonstrated that abscisic acid (ABA) pretreatment significantly influences transcriptomic reprogramming in plants, facilitating earlier activation of stress pathways and enhanced recovery from drought stress. The observed differences in gene and transcript expression dynamics between ABA-pretreated plants and those subjected to drought stress alone underscore the regulatory mechanisms modulated by ABA.

### ABA impacts stomatal conductance and photosynthesis

Stomatal conductance plays a pivotal role in modulating plant water status. Our results showed that ABA pretreatment led to stomatal closure, a well-known initial response to water deficits. ABA activates an ABA-dependent cascade of reactions, leading to reduced turgor pressure in guard cells and stomatal closure [35, 36, 41]). ABA-induced stomatal closure helps plants to minimize water loss via transpiration and conserve water during drought conditions [25]. The pronounced response of stomata to ABA is associated with a drought-tolerant phenotype [30, 49]. Notably, even after a two-week interval post-ABA treatment, the stomata remained partially closed, suggesting a prolonged effect of ABA in modulating stomatal behavior. A similar effect was observed in drought-treated plants, highlighting the role of ABA in the earlier activation pathways in response to further exposure to drought stress (Fig. 2). Our findings regarding the chlorophyll content of ABA pre-treated barley plants further highlight the protective role of ABA. Chlorophyll, which is the primary pigment responsible for photosynthesis, is sensitive to drought stress. Many studies have reported a drought-induced decline in the chlorophyll content ([46], [74], [58]. Drought affects the stability of chloroplasts, induces chlorophyll photooxidation, and inhibits the expression of chlorophyll biosynthesis genes is inhibited [37],[20]. A decline in the chlorophyll content, as observed in drought-treated plants, can hinder photosynthetic efficiency. However, ABA pre-treated plants exhibited higher chlorophyll content under drought conditions than only drought treated plants, suggesting that ABA might protect chlorophyll molecules from degradation or potentially stimulate their biosynthesis under water-deficit conditions. ABA is known to activate the genes responsible for chlorophyll breakdown [19, 68]. However, it have been shown that low ABA concentrations have a protective effect on the chlorophyll content in response to drought [67],[4]. Furthermore, our photosynthetic data, particularly regarding photosystem II activity, further support the protective effects of ABA under drought conditions. ABA pre-treated plants maintained efficient photosynthesis during drought, which could be attributed to a mechanism stimulated by ABA to protect the photosynthetic apparatus from damage (Fig. 2). The protective role of ABA on photosynthesis in response to drought has been previously shown in other studies [2, 6, 23]. They observed more pronounced photosynthesis inhibition in response to drought-only treatments than in ABA-pretreated plants under drought conditions. Low concentrations of ABA can alleviate drought-induced changes in the photosynthetic apparatus. One of the proposed mechanisms is the involvement of ABA in the xanthophyll cycle, which ensures the removal of excess energy from the photosystem and maintains the chlorophyll content, photosynthetic efficiency, and chloroplast ultrastructure non-disturbed [21, 23, 45]. The differential expression of genes related to photosynthesis in ABA pre-treated plants further underscores the role of ABA in bolstering the photosynthetic machinery under drought conditions. The transcriptomic changes observed in the photosynthesis-related genes further illustrated the adaptive mechanisms induced by ABA pretreatment. The upregulation of these genes during drought and their subsequent downregulation during recovery suggests a dynamic regulatory process aimed at optimizing resource allocation. This regulation ensures that photosynthetic efficiency is maintained during stress, while conserving resources during recovery, which is critical for plant survival and growth. Previous studies have reported similar regulatory patterns in photosynthetic genes under stress conditions [9, 34].

### Transcriptomic insights reveal ABA pretreatment role in activation of earlier molecular responses to drought

ABA pretreatment primes plants for a more robust response to drought stress, as evidenced by the distinct grouping in PCA and the quicker recovery of ABA-pretreated plants (AD3) compared with drought-only treated plants (D3). This early activation of stress pathways is likely due to ABA-induced modulation of gene expression, particularly the upregulation of genes involved in abscisic acid response, water stress, and DNA modifications. These findings align with those of previous studies that highlighted the role of ABA in priming plants for stress resistance through transcriptional reprogramming [14, 59].

Differential expression analysis revealed that ABA pretreatment led to a higher number of differentially expressed genes (DEGs) and transcripts (DETs) during both the stress and recovery phases. Notably, genes associated with chromatin and DNA modifications were significantly regulated, suggesting that ABA pretreatment influences chromatin remodeling and transcriptional regulation, thereby enhancing stress tolerance. These results are consistent with previous reports indicating that chromatin modifications play a crucial role in stress memory and adaptation [31]. This suggests that ABA pretreatment might prepare the plant at a molecular level, priming specific pathways or processes and ensuring a more resilient response when exposed to drought stress. Epigenetic modifications such as DNA methylation and histone modifications have been implicated in plant stress responses, indicating that they might play a role in ABA-mediated drought tolerance. Drought-related epigenetic changes often affect ABA-related genes, *NCED3, PYLs* and *ABI4*. In response to stress, the status of DNA methylation and/or histone acetylation/methylation is modified to induce the expression of ABA-related genes [16, 29, 42]. The role of DNA modifications such as methylation in stress responses and memory has been documented in plants. Stress-induced changes in DNA methylation can be transferred to the next generation to enable more efficient stress [1, 11]. ABA may instigate these modifications, providing a memory of the stress that could benefit subsequent exposure. It has to be underlined that the ABA pathway component, ABI3, was already shown to induce epigenetic modifications via histone demethylase, JUMONJI-C domain-containing protein 30 (JMJ30) [72].

A significant discovery in our study was the prominent role of alternative splicing (AS) in mediating plant responses to drought stress following ABA pretreatment. Our data revealed that splicing is a critical driver of gene expression changes, with numerous differentially alternatively spliced genes (DAS) are enriched in RNA metabolism processes. This enrichment underscores the importance of AS in the regulation of RNA stability and processing under stressful conditions. Similar findings have been reported, in which AS contributes to stress adaptation by generating transcript isoforms with distinct functions [18, 63]. The increased prevalence of isoform switching in ABA-pretreated plants under drought stress indicates a sophisticated layer of post-transcriptional regulation, potentially allowing for the production of functionally diverse protein isoforms. This mechanism may enhance the ability of plants to adapt to changing environmental conditions by fine-tuning their gene expression [52, 65].

### Endogenous ABA signaling and drought response

Our RT-qPCR results provide a comprehensive view of the endogenous ABA signaling cascade in response to drought. The differential expression of genes involved in ABA biosynthesis, catabolism, and signaling suggests fine-tuned regulation of the ABA pathway under drought stress. Our results indicated an upregulation of genes such as *HvBG8* in plants pre-treated with ABA, hinting at the existence of a feedback mechanism where ABA pretreatment could enhance the plant's ability to respond more effectively to drought stress. *HvBG8* is of particular interest, because it is involved in ABA deconjugation, which is pivotal for the release of active ABA in response to stress (Seiler et al. 2014b; [39]). This suggests that ABA pre-treatment may prime plant endogenous ABA signaling, enabling a more potent and rapid response to water deficit conditions, potentially leading to enhanced drought tolerance.

For example, genes involved in ABA biosynthesis, such as *HvZEP1* and *HvNCED1*, are crucial for maintaining a delicate balance between ABA synthesis and catabolism, potentially affecting plant drought tolerance.

Our findings suggest that ABA pretreatment might stimulate the ABA pathway at multiple levels, from perception of the ABA signal to activation of ABA-responsive genes. Modulation of the expression of ABA receptors, kinases, and transcription factors, such as *HvPYL5* and *HvSnRK2.1*, reflects the complexity of the endogenous ABA signaling network in response to drought stress. The observed differential expression of these genes at different time points further underscores the temporal dynamics of ABA signaling under drought conditions, warranting further exploration to comprehend time-dependent regulatory mechanisms. The increased expression of ABA-responsive genes, such as those encoding LEA and *HvA22*, in ABA pre-treated plants suggests a potential ABA-induced enhancement of stress response mechanisms. Such enhancements may improve stress tolerance, allowing the plant to maintain physiological processes, such as photosynthesis, under unfavorable conditions, as the protective role of LEA in photosynthesis has been confirmed [64]. This is particularly crucial for crop productivity in water-limited environments where ABA-induced enhancements in stress responses can mitigate yield losses due to drought.

### Influence of ABA on yield parameters

Drought imposes severe stress on plants, disrupting multiple yield parameters and reducing the overall productivity. Our findings demonstrate that ABA pre-treatment can alleviate some of the detrimental effects of drought, offering a potential strategy to improve plant resilience and maintain yield stability under water-limited conditions. Specifically, ABA pretreatment helped sustain critical yield components, such as the number of spikes, which remained comparable to that of the control plants. These results highlight role of ABA in preserving crop productivity under drought stress conditions. Additionally, the conservation of seed area in ABA-pretreated plants underscores ability of ABA to maintain seed quality, which is critical for the overall yield stability. However, ABA pre-treatment also led to an increased number of sterile spikes, suggesting a trade-off between enhanced stress tolerance and reproductive success. This trade-off underscores the complexity of ABA role in drought responses, where its protective effects on photosynthesis and growth may come at the cost of reproductive development. These findings emphasize the need for a more refined understanding of the mechanisms underlying the effects of ABA on plant fertility, particularly in the context of optimizing ABA use for drought resilience. To address this trade-off, we conducted a subsequent experiment using adjusted ABA application strategies, focusing on lower ABA concentrations and earlier timing. Barley plants treated with 25 µM ABA at the tillering stage showed significant improvements in yield parameters, including thousand-grain weight (TGW) and seed weight per plant. Importantly, the number of sterile spikes in this treatment remained comparable to that in drought-only plants, indicating that precise adjustments to ABA dosage and timing can mitigate its negative effects on fertility while preserving its positive impact on yield. These findings suggest that strategic ABA application can enhance specific yield components and improve photosynthetic efficiency under drought stress. Although the direct application of ABA may be economically impractical for large-scale use by breeders, this study provides valuable insights into the significance of early activation of stress-responsive pathways and associated molecular mechanisms in enhancing drought resilience. These findings can inform breeding programs that focus on developing barley varieties with naturally enhanced endogenous ABA production or improved regulation of stress-response pathways. Such strategies could offer cost-effective and sustainable alternatives to exogenous ABA application, ensuring stable crop productivity under drought conditions, while reducing reliance on expensive treatments.

## Conclusions

This study demonstrates that ABA pretreatment significantly enhances drought resilience in barley by modulating physiological, molecular, and transcriptomic processes. ABA application triggers stomatal closure, preserving water balance while maintaining chlorophyll content and photosynthetic efficiency under drought stress. The protective effect on the photosynthetic apparatus, including PSII performance, highlights ABA role in mitigating the adverse effects of water deficiency on primary metabolism. At the molecular level, ABA pre**-**treatment facilitates earlier activation of stress-response pathways, as evidenced by distinct gene expression and alternative splicing patterns. Notably, ABA pre-treatment promotes a dynamic transcriptome reprogramming response, including:

- enhanced alternative splicing and isoform switching, which fine-tune gene expression and post-transcriptional regulation under stress
- upregulation of genes associated with chromatin remodeling, DNA methylation, and RNA metabolism, revealing a critical role of epigenetic and post-transcriptional modifications in ABA-mediated stress adaptation.

These processes enable ABA-pretreated plants to transition more efficiently from stress response to recovery, accelerating repair mechanisms and restoring metabolic homeostasis following rewatering. Importantly, ABA pre-treatment sustains key yield components under drought stress, including spike number and seed area, while mitigating the decline in photosynthetic efficiency and overall plant performance. However, a trade-off was observed in reproductive outcomes, with increased sterile spikes under specific ABA application regimes. Adjusting the timing and concentration of ABA treatment, such as applying lower doses (25 µM) at the tillering stage, effectively mitigated this negative effect, improving yield parameters like thousand-grain weight (TGW) and seed weight per plant. While direct ABA application may be impractical for large-scale agricultural use, this study provides crucial molecular insights for alternative strategies. Breeding programs can leverage these findings to develop barley genotypes with enhanced endogenous ABA biosynthesis, improved regulation of ABA-responsive pathways, and efficient transcriptome plasticity. Furthermore, the identified ABA-induced stress mechanisms pave the way for cost-effective agronomic interventions, including hormone analogs, biostimulants, and optimized irrigation practices that mimic ABA beneficial effects.

## Supplementary Information

Supplementary Material 1. Table S1. Transcriptomic analysis at gene and transcript levels List of all identified DEGs, DETs, and DAS across treatments.

Supplementary Material 2. Table S2. Gene Ontology analysis of genes classified in Clusters 4 and 9 identified hierarchical clustering of DETs in the AD2 variant.

Supplementary Material 3. Table S3. Transcripts Per Million (TPM) values obtained during the RNA-seq analysis of ABA-related genes were studied using RT-qPCR.

Supplementary Material 4. Figure S4. Example of grain scans of barley from each of experimental variants: C, A, AD, and D, obtained using GrainScan (A). Grain parameters of barley from each of experimental variants: C, A, AD, and D, obtained using GrainScan (B).

## Data Availability

All raw transcriptomic data used in this study can be found in the following repositories: ArrayExpress and can be accessed with E-MTAB- 14333.

## Abbreviations

ABA: Abscisic acid
ABS/CS: Absorption flux per cross section
DAS: Days after sowing
DEGs: Differentially expressed genes
DI_0_/CS: Dissipation flux per cross section
ET_0_/CS: Electron-transport flux per cross section
GO: Gene ontology
PI_ABS_: Performance index
RC/CS: Reaction centers per cross section
ROS: Reactive oxygen species
TF: Transcription factor
TGW: Thousand Grain Weight
TPM: Transcript per million
TR_0_/CS: Trapping flux per cross section
VWC: Volumetric water content
