# Supplementary material for: ABA-induced alternative splicing drives transcriptomic reprogramming for drought tolerance in barley
Source: BMC Plant Biol. 2025 Apr 8;25:445. doi: 10.1186/s12870-025-06485-y (PMC11977895; doi:10.1186/s12870-025-06485-y)

**Supplementary Material S4**.

**(a) Example of seed scans of control barley and barley treated with ABA, drought and ABA+drought obtained using GrainScan.**


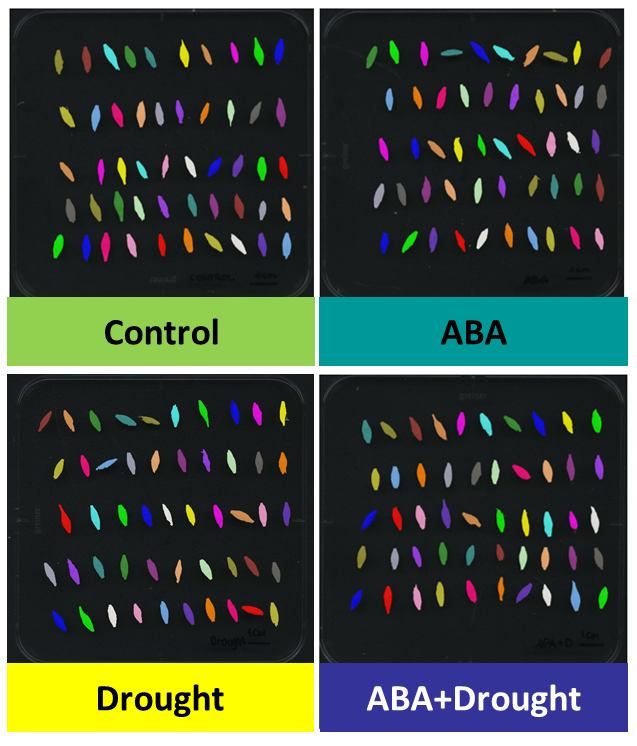


**(b)** **Seed parameters of control plants and plants treated with ABA, drought and ABA+drought obtained using GrainScan.**

**(A)** Seed area, **(B)** seed circuit, **(C)** seed length, **(D)** seed width, **(E)** lightness of the grain colour (0-100) - grain Ch1, **(F)** redness, green (negative values) or magenta (positive values) - grain Ch2 and **(G)** yellowness, blue (negative values) or yellow (positive values) - grain Ch3 (Whan et al. 2014). One-way ANOVA (P ≤ 0.05) followed by Tukey HSD test (P ≤ 0.05) was applied to identify differences between analysed variants of the experiment. Statistically significant differences (P ≤ 0.05) are indicated by different letters.


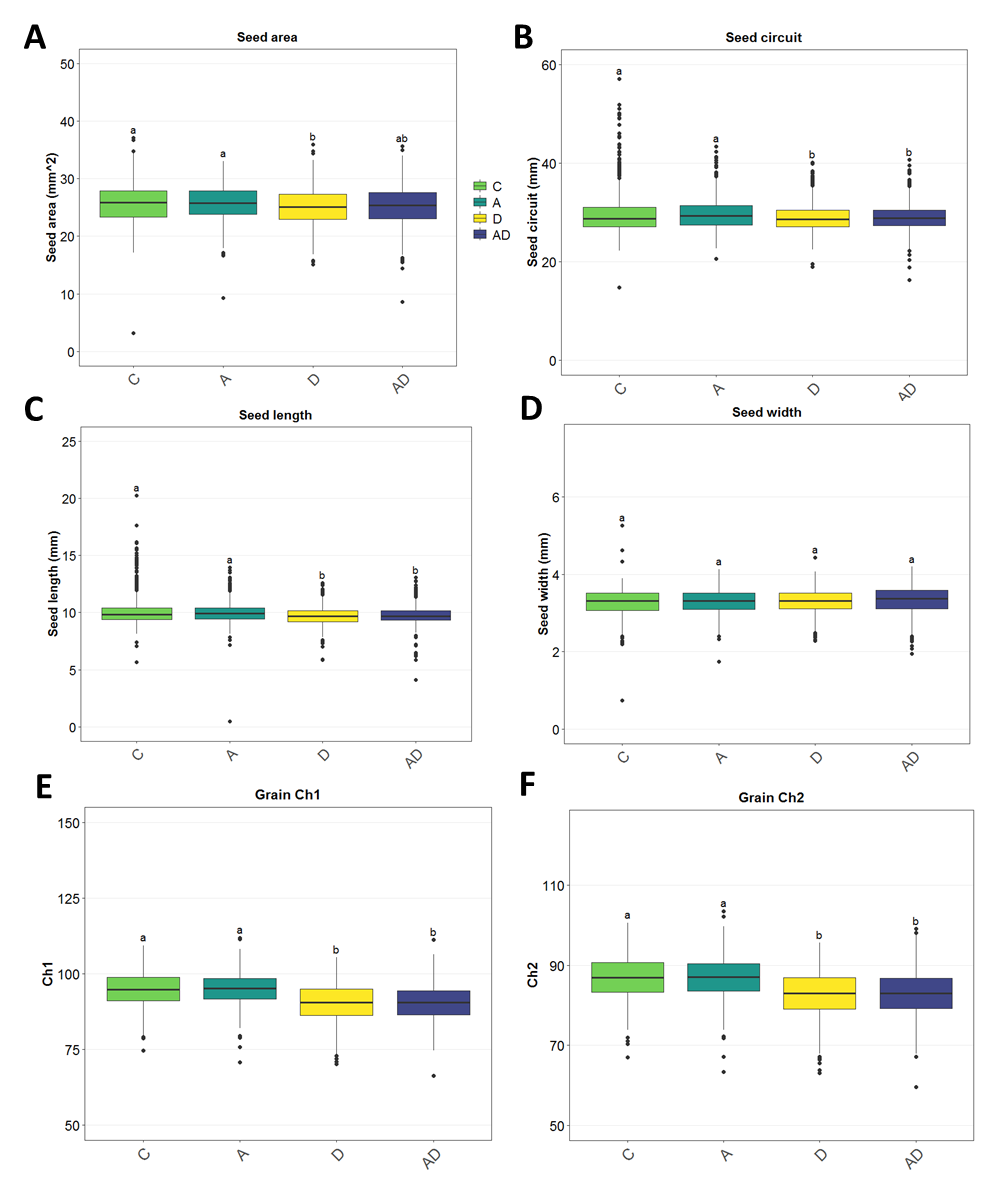

Supplement: Supplementary file 4 — Supplementary Material 4. Figure S4. Example of grain scans of barley from each of experimental variants: C, A, AD, and D, obtained using GrainScan (A). Grain parameters of barley from each of experimental variants: C, A, AD, and D, obtained using GrainScan (B). [file 12870_2025_6485_MOESM4_ESM.docx]
